# Supplementary material for: Re-imagining health research to include the voices of justice-impacted individuals
Source: PLOS Glob Public Health. 2026 Mar 3;6(3):e0006069. doi: 10.1371/journal.pgph.0006069 (PMC12956120; doi:10.1371/journal.pgph.0006069)
Supplement: S1 Table — This checklist was given to advocacy partners as an online survey to aid in their recruitment for eligibility and collect the demographic data of potential participants. (DOCX) [file pgph.0006069.s001.docx]

## Supplemental Table 1. Recruitment Checklist

| **Inclusion criteria** | **Category details** | **Recommended recruitment number per Focus Group** |
| --- | --- | --- |
| Incarceration Experience | Has ever been incarcerated and not currently incarcerated | 10 |
| Age | Must be 18 years or older | 10 |
|  | | |
| **Demographic questions for participants** | | |
| Race/Ethnicity  Which race/ethnicity category best describes you? | American Indian or Alaska Native | 1 |
|  | Asian | 1 |
|  | Black, African or African American | 3 |
|  | Hispanic, Latino or Spanish | 2 |
|  | Middle Eastern or North African | 1 |
|  | Native Hawaiian or Other Pacific Islander | 1 |
|  | White | 3 |
|  | More than one (2+) / Multi-Ancestry | 1 |
| Gender  Which category best describes you? | Male | 9 or less |
|  | Female | 1 or less |
|  | Others (transgender, gender neutral, non-binary, agendaer, pangender, genderqueer, two-spirit, third gender, all, none, or combination of these) | No recommendation |
|  |  |  |
|  |  |  |
| Income  What is your annual household income? | Less than $10,000 | No recommendation |
|  | $10,000-$24,999 | No recommendation |
|  | $25,000-$34,999 | No recommendation |
|  | $35,000-$49-999 | No recommendation- |
|  | More than $50,000 | No recommendation- |
|  | | |
| Email | After participating in the focus group, we will send the electronic gift cards after the focus group using this email address. | Email: |
