# Supplementary material for: Re-imagining health research to include the voices of justice-impacted individuals
Source: PLOS Glob Public Health. 2026 Mar 3;6(3):e0006069. doi: 10.1371/journal.pgph.0006069 (PMC12956120; doi:10.1371/journal.pgph.0006069)
Supplement: S1 Text — This section includes all recruitment communications to participants. (DOCX) [file pgph.0006069.s002.docx]

## Recruitment Communications

### Initial Recruitment Flyer

Note: there were also recruitment emails, phone calls, social media posts, and text messages with similar content.

Title: Focus Group for Anyone that has been Previously Incarcerated

Alt Title: Previously incarcerated persons: Focus Group

- We Want to Hear from You!
- 75-120-minute virtual Zoom meeting
- For individuals age 18+ who have ever been imprisoned within the United States
- If interested, contact <Contact person, Advocacy Organization, Contact person, Organization phone number>
- Space is limited
- All participants will receive a $70 electronic gift card [note, compensation will not be emphasized by the flyer’s formatting]
- Join us for a focus group discussion about life after incarceration and your perspective on research.
- *Contact name and number will be listed individually by location dependent on partner

| **Location** | **Contact Person** | **Phone number** |
| --- | --- | --- |
|  |  |  |
|  |  |  |
|  |  |  |

### Recruitment Script

Hello, thank you for your interest in participating in our study. My name is [Contact Person Name] and I am assisting a research study from Scripps Research Translational Institute to better understand any reasons why individuals that were formerly incarcerated would be willing and able to participate in research (ex. perspectives, time/availability, other activities or priorities). I would like to provide you with some information about the study and answer any questions you may have. Would you be interested in hearing more about participating in this study?

If no - Thank you for your time. Have a nice day/evening.

If yes - Great! Let me give you an overview of the study. If you are interested in participating, we will ask a few screening questions to check eligibility. If you are eligible and are selected, you may receive an invite to a focus group interview. This session will involve around 9 other people and will last about 75-120 minutes. In the focus group, we are interested in hearing your thoughts and experiences. We will ask you things like what you think research should do and what problems it should solve. The discussion might touch on your experiences before and after your time in incarceration and how that might influence your interest in research and science. The session will be audio-recorded on Zoom, but only for research purposes. We will keep all the information and recordings in a secure research database managed by trained staff. Plus, to protect your identity, you will get to pick pseudonyms (fake names). Your participation is completely voluntary and you can withdraw at any time without penalty. Based on the information provided, I’d like to confirm that you understand the details and agree to participate. Are you still interested in taking part?

If no - Thank you for your time. Have a great day/evening.

If yes - Great! Thank you for confirming your interest. We will proceed with next steps and provide you with further instructions.
