# Supplementary material for: Re-imagining health research to include the voices of justice-impacted individuals
Source: PLOS Glob Public Health. 2026 Mar 3;6(3):e0006069. doi: 10.1371/journal.pgph.0006069 (PMC12956120; doi:10.1371/journal.pgph.0006069)
Supplement: S2 Text — This contains the IRB approved script used to guide focus group discussions. (DOCX) [file pgph.0006069.s003.docx]

### Focus Group Script

**1**. **Introduction:**

**Moderator**: Good evening, everyone, and thank you all for joining us today for this important discussion. My name is [Moderator name] with [Focus Group organization name] and I'll be your host and facilitator for today's session. We are working with Scripps Research to learn about how to engage people with research programs. We truly appreciate your time and willingness to share your thoughts and experiences with us. We ask that you keep your cameras off to protect your anonymity with this focus group.

I'd like to extend a warm welcome to each one of you. This discussion aims to explore your overall knowledge and thoughts on research and science, what would make you want to participate in research (or not), and what barriers or challenges you may have that would make it hard for you to participate if you did want to. Your perspectives as returning citizens are invaluable, and we're excited to hear your insights.

We understand that you may have various feelings and opinions about this focus group, and our goal today is to create an open, respectful, and safe space where everyone feels comfortable sharing their thoughts. Your participation will help inform and improve the program's outreach and impact, so please know that your input is extremely valuable.

For the purpose of this focus group, we will be audio recording only, Zoom will provide a transcript of responses, and I will be taking notes. We will destroy the recordings, transcript and notes as soon as we have completed the report. We will not use your real names in preparing the notes or report, we will use the pseudonyms that everyone selected during recruitment for the notes and use a unique number identifier for the report.

Again, thank you for being here, and we're looking forward to an engaging and productive discussion. Let's begin by introducing ourselves.

**2**. **Icebreaker/Introduction of Participants**

- **Weird Week.**
  - We will start by introducing ourselves using our pseudonyms
  - What is the weirdest/ strangest thing you saw/ heard/ did this week?

**[Moderator]:** Before we dive into our discussion, I'd like to take a moment to go over some Ground Rules.

- **Ground Rules**
  - Respect the time of the other participants and the moderator.
  - Make sure you have a stable internet connection and a quiet space with minimal distractions.
  - Ensure that your microphone is working properly.
    - Reminder: Make sure all cameras are turned off.
  - Listen attentively to the other participants and do not interrupt when someone is speaking.
  - Speak clearly and concisely to make sure your points are understood by others.
  - Please stick to the topic at hand and avoid discussing unrelated subjects.
  - Avoid using inappropriate language or making derogatory remarks about other participants.
  - Stay focused and engaged during the discussion, and avoid multitasking or other activities that could distract you.
  - Keep the confidentiality of the information shared during the focus group session.
  - Follow the moderator's instructions and guidelines regarding the structure and format of the focus group.

Please know that your participation today is completely voluntary, and we will respect your confidentiality throughout the process. The information you share will be anonymized and combined with input from other focus groups to generate a comprehensive understanding of what your thoughts are on research, what would make you want to participate in research (or not), and what barriers or challenges you may have that would make it hard for you to participate if you did want to.

Once again, thank you for your participation, and we're looking forward to hearing your valuable insights. Now, let's begin our discussion [BEGIN ZOOM RECORDING].

**3. Discussion Topics: choice of questions below**

1. Objective 1: Exploring overall knowledge and perspectives of returning citizens regarding research and science.
   1. The purpose of health research is to gather information, and test it so that we can find solutions and solve problems for health treatments and care. What do you think research should do? What problems do you think it should solve?
   2. What do you think of when you hear "Research program"?
   3. How interested would you be in knowing your health risk factors (physical, mental)?
   4. How interested would you be in knowing your ancestry? To know where your family came from?
2. Objective 2: Determining barriers that prevent returning citizens from participating in research (ex. perspectives on health, time/availability, other activities or priorities).
   1. How supported do you feel by your community and resources?
      1. Justification: To understand potential availability for research participation. Are those that are receiving community resources more likely to be open to research participation?
   2. What do you spend the most time on a regular basis working towards?
      1. Justification: To understand time constraints by other activities.
   3. Have you ever participated in any research in the past? If yes, what were the reasons for participating? If not, what were the reasons for not participating?
   4. How important is your physical health and wellness to you?
   5. How important is your mental health to you?
   6. Are there any activities you would like to participate in?

**4. Follow-up Questions (10-15 min)**

1. What are some specific actions or habits that you take to maintain your physical health and wellness?

2. What specific activities bring you the most joy or satisfaction?

3. Is there something you have always wanted to try to learn, but have not had the opportunity to do so?

- Are there any educational or vocational programs you would be interested in pursuing?

**5. Wrap-Up**

1. We appreciate your time and insights today.
2. We will provide you with a list of resources from [Advocacy Partner name] that may be helpful for returning citizens. You will receive an email with a $70 electronic gift card within 72 hours of the focus group.
3. Is there anything else you would like to add or any questions you have for us?
4. Thank you again, and have a great day!
