# Supplementary material for: Re-imagining health research to include the voices of justice-impacted individuals
Source: PLOS Glob Public Health. 2026 Mar 3;6(3):e0006069. doi: 10.1371/journal.pgph.0006069 (PMC12956120; doi:10.1371/journal.pgph.0006069)
Supplement: S3 Text — This section provides more details and quotes from the focus group participants that aided the analysis of the results. (DOCX) [file pgph.0006069.s004.docx]

## More Detailed Focus Group Analysis

### Objective 1: Exploring overall knowledge and perspectives regarding research and science.

70% of the participants responded that research should lead to an improvement in quality of life. Many saw research as a tool for solving concrete health problems, suggesting a pragmatic view of research as a means to develop treatments and improve healthcare outcomes for the community. The following are quotes from the participants on this topic, adapted to maintain the grammatical integrity and remove identifiable linguistic features such as regional or accented speech:

- “I think health research, number one, should achieve a kind of improvement in the previous rediscovery of health. Discovery, and then try to better the situation, health conditions of the end users. Because, researching health and then getting more improvement will help develop a lot of things. And then we help eradicate a lot of health imbalances in society.” Participant P1-FG1-P3
- “It's just important to make sure that you gather as much data from as many socioeconomic groups as possible and to do it over a long period of time. [The goal is] to get accurate data to allocate resources appropriately, prioritize those resources, and, of course, ensure that the right policies are in place.” Participant P2-FG2-P1
- “Health research should not just be a process of trying to get to solutions, but it should also be a process of analysis of that process to figure out how we get to certain places. So, not just analyzing the result, but analyzing the process in which we reach a result.” Participant P2-FG1-P7

A majority of participants, particularly from the 40-55 and 55+ age groups, shared a concern that research would treat them as guinea pigs and lab rats. The younger participants held generally more positive responses including investigating and solving problems, and improvements for society. This trend continued throughout the focus groups, as the over 40 age groups were generally more hesitant to participate in different aspects of research than the under 40 groups. The following are quotes from the participants on this topic, adapted to maintain the grammatical integrity and remove identifiable linguistic features such as regional or accented speech:

- “I think of lab rats, I think of guinea pigs. Anytime in my mind doing medical research, you're always tweaking that to make it have desired effects. But the first effects are going to be the most harmful.” Participant P4-FG1-P5
- “All the way back to the Tuskegee research, [research] always triggers. From lived experience, historical experience, it's been something that we took caution with. We're the last ones to want to try anything experimental or anything like that.” Participant P1-FG2-P3

When asked, all participants found their physical health to be important. 90% of participants were interested in knowing their health risk factors. The participants put their longevity in terms of “time spent with family” and “furthering their quality of life.” There was a consensus that mental health was important. Many of the participants shared that they faced challenges to their mental health while incarcerated (some for systemic reasons, some for anecdotal reasons, and some due to both) and the importance of developing and maintaining strong mental health. 93%of the participants responded that exercise, focusing on mental health and diet were the main routines they use to ensure well-being. Also, 85% were interested in learning about their genetic ancestry and any impact this has on their health. Below are quotes on this topic, adapted to maintain the grammatical integrity and remove identifiable linguistic features such as regional or accented speech:

- “Just being able to, you know, give people the proper information about what's going on, so they don't have to feel left out at any level and understanding of what they possibly could do to live a better life, mentally or physically. You know, especially after being incarcerated, or even men of color. It's been taboo for a long time to deal with certain situations and issues, not understanding that it was mental health.“ Participant P5-FG1-P4
- “I definitely want to know. And I want to know more about it. A lot of times, I mean, just using an example, you go to [health care provider], you know, you get checked out, and they, you know, diagnose you with whatever they diagnose with. Let's just say high blood pressure. They give you a handout, and it says, "Here, you know, here's some information on that." Well stop and explain that stuff to me. Let's talk about this for a second. I know they've got the patient quota, I know they've got a lot of stuff they have to do, but stop and explain some of that stuff, and I think there's a better chance that we would actually make a decision to improve our lifestyle.” Participant P4-FG1-P2
- “I think that knowing your ancestry is very important, especially in the conversation that we've been having about knowing your health history. If you're connected with your ancestors, you can kind of beat some of the medical issues before they come up. And just knowing where you come from is very key to identifying different illnesses and other problems that are in your genetics, that's in your bloodline. So I think that's very important for us to know where we come from, who we come from.” Participant P3-FG1-P2

### Objective 2: Determining barriers that prevent returning citizens from participating in research (e.g., perspectives on health, time available to contribute to research versus other activities and priorities)

66% of participants felt supported by their community. Family, friends, advocacy organizations were the most common sources of support during and after incarceration. Below are quotes on this topic, adapted to maintain the grammatical integrity and remove identifiable linguistic features such as regional or accented speech:

- “Over the past 15 years, I never felt supported until this last time when I did the 5 years in prison. But the support was there, I had to reach out and go get it. I had to put the work in, do the research, make calls, and show up. I really had to put myself out there and be vulnerable, and humble myself. But this time, I have really felt supported, and it's done me well.” Participant P5-FG2-P2
- “I feel supported by my family and friends, and I'm so grateful and humble. The other support that I see is from the perspective of a program that I'm currently in with my job and everything. If it wasn't for that, I couldn't even tell you where I would be at.” Participant P2-FG1-P1
- “My biggest support and only support that I received was from the organization that I now work for. They were the ones that gave me home, funding, job, therapy, everything in one package, and so I can say yes. I was supported.” Participant P4-FG2-P4

Participants who answered that they felt supported after their incarceration terms were more open to the idea of research participation. This suggests that community engagement could be a significant factor in facilitating involvement in research. Below are quotes on this topic, adapted to maintain the grammatical integrity and remove identifiable linguistic features such as regional or accented speech:

- “I guess it made me more confident to participate because I would have good advisors around me. And I know that at the end of the day, if the people you're doing research with are good people, then yeah, it's going to help.“ Participant P1-FG1-P2
- “The child that's not embraced will burn the village down. [If ]You know you're fed, you have shelter. You also have the resources; whether that's internet, a computer, to have the ability to participate in this kind of stuff [referring to research].” Participant P3-FG2-P3
- “If the research is being conducted by an advocacy organization or an organization that is interested in implementing change, then, of course, I'm amenable to participating in such things. So, if there is an organization that says, "Hey, we want to investigate this situation. We want to get data on this, on how further marginalizing and denigrating the reentry population not only harms that population but harms the fabric of our society as a whole," then I absolutely want to be involved with that. Participant P5-FG2-P5

When asked how participants spend their time. The most common answer (43%) was work. Other responses included health (15%) and family (13%). Participants spoke about going back to school, such as finishing their high school education, and ensuring their own longevity to take care of their family. These activities were prioritized over research participation and weighed against the potential time commitments to and compensation from the research study. This suggests that accessibility, narrower time commitments and higher financial incentives can garner greater interest in future research participation. The majority of the participants (59%) had previously participated in research, perhaps because they were already connected with an advocacy organization that may bring these opportunities to them. There were no significant demographic differences when accounting for age, race or gender of the participant groups. Participants shared various reasons for participation in research, including personal benefits and curiosity, but the most popular answer was the financial incentive. The opportunity costs of time and money when participating in research versus working was weighed throughout the focus groups by several of the participants. Participants cited a lack of opportunity (incarceration, schedule, not aware of other research opportunities) as their primary reason for not participating in research.
