# Supplementary material for: Re-imagining health research to include the voices of justice-impacted individuals
Source: PLOS Glob Public Health. 2026 Mar 3;6(3):e0006069. doi: 10.1371/journal.pgph.0006069 (PMC12956120; doi:10.1371/journal.pgph.0006069)
Supplement: Attachment — Submitted filename: RC - response to editor PLOS GPH.docx [file pgph.0006069.s005.docx]

Editor’s feedback on submission PGPH-D-25-00078, and author response.

Thank you for submitting your manuscript reporting on a study exploring the perspectives of individuals with prior justice involvement regarding research and science and identifying barriers to their participation in research.

After a thorough review of your submission, I regret to inform you that it does not meet the journal’s standards for publication in its current form:

- The methods section is significantly underdeveloped. With only four short paragraphs, it lacks the necessary detail to meet the standards for qualitative research, such as those outlined in the COREQ guidelines.

Author response: We added substantial information regarding the risks and mitigations undertaken to protect participant privacy, as well as descriptions of each step of the data analysis.

- There is insufficient information provided about the characteristics of the study participants, which limits the reader's ability to contextualize and evaluate the findings.

Author response: In addition to the participant demographics outlined in table 1, we added the physical locations of each community partner.

Should you decide to resubmit, providing more detailed methods, including adherence to established reporting guidelines and comprehensive participant information, will enhance the manuscript's impact and alignment with the journal's standards.
