# Supplementary material for: Re-imagining health research to include the voices of justice-impacted individuals
Source: PLOS Glob Public Health. 2026 Mar 3;6(3):e0006069. doi: 10.1371/journal.pgph.0006069 (PMC12956120; doi:10.1371/journal.pgph.0006069)
Supplement: Attachment — Submitted filename: RC Response to Reviewers August 29.docx [file pgph.0006069.s006.docx]

Response to Reviewers August 29, 2025 for the manuscript entitled:

Re-imagining health research to include the voices of justice-impacted individuals

1. As you have noted that you cannot publicly share a deidentified data set or provide a non-author point of contact to request access to these data due to their destruction, we would appreciate if you could please ensure that you have addressed the following items in your Data Availability statement:

a) Discuss the reason why the data cannot be made publicly available and why the authors must be the points of contact for fielding data access requests.

b) List multiple authors as points of contact and include their contact information (i.e., email address).

c) Provide additional details on how you will ensure persistent or long-term data storage and availability.

d) Specify in your Data Availability statement who will have access to the data and the criteria you will use to consider requests (e.g., the limitations of data sharing as outlined in the IRB approved protocol or patient consent form).

e) Include the following text in your Data Availability statement: “Although the authors cannot make their study’s data publicly available at the time of publication, all authors commit to make the data underlying the findings described in this study fully available without restriction to those who request the data, in compliance with the PLOS Data Availability policy. For data sets involving personally identifiable information or other sensitive data, data sharing is contingent on the data being handled appropriately by the data requester and in accordance with all applicable local requirements.”

We will update your Data Availability statement on your behalf to reflect the information you provide. As with all submissions, we are committed to data availability; if concerns are brought to our attention after publication, we will follow up to ensure appropriate availability of the data and will update the scientific record as needed.

Finally, for future research, note that as there are growing expectations for making underlying data available with published research and an increasing number of repositories for storing sensitive data, we would recommend consulting with your institutional research data management services or a data librarian to consider what other data management options might be available.

Author response: Thank you for this thoughtful feedback. We update the data availability statement and will keep this in mind for future work.

2. Please ensure that you refer to Figure 1 in your text as, if accepted, production will need this reference to link the reader to the figure.

Author response: We added a reference to Figure 1 in the main text.
