# Supplementary material for: Re-imagining health research to include the voices of justice-impacted individuals
Source: PLOS Glob Public Health. 2026 Mar 3;6(3):e0006069. doi: 10.1371/journal.pgph.0006069 (PMC12956120; doi:10.1371/journal.pgph.0006069)
Supplement: Attachment — Submitted filename: RC response #3 to PLoS Global Public Health.docx [file pgph.0006069.s007.docx]

Reviewer’s feedback on submission PGPH-D-25-00078, and author response.

December 11, 2025

Reviewer #2: This is an interesting project, on an important and interesting topic – capturing the voice of individuals who ordinarily lack a platform to be heard – this is commendable! However, I hope my comments below, help to strengthen this work further.

Author response: Thank you, for this commendation.

General style and points

The paper could benefit from improving conciseness in places – e.g., in the study design section ‘‘The information shared about the moderator was their name, that they were moderating for the respective Advocacy Organization and that the study was being conducted by Scripps Research’ – maybe reword to something like ‘Participants were shared the following information: moderators name, their role, and that Scripps Research was conducting the study’. It would be worth reading the paper with a similar approach to other sections too.
Author response: Thank you for the recommendation, a duplicative sentence about the study design was removed on page 6 and updated on page 7 to say “Participants were given the following information: the moderator’s name and role, that Scripps Research was conducting the study, the study objectives and measures the study took to maintain the participant’s privacy (e.g destruction of transcripts after completion of the study report, use of pseudonyms).” Similar edits were made on page 7 for conciseness.

Pg5 there is a double ‘..’ after ‘(see Table 1 Advocacy Organization Partners)..’ – this should be singular
Author response: Thank you, this has been updated.

Ethics
The recruitment section suggests that participants were recruited by advocacy partners via phone calls – this seems a little targeted? It would be worth demonstrating how this was still on a voluntary, fully-informed, basis – this seems to stand out from the other methods e.g., text messages, social media posts – where people can choose / volunteer to get involved. Maybe explanation of this is required.
Author response: The Advocacy organizations used their community’s preferred method of communication. We added the following context to the manuscript “ In general, the advocacy organizations had existing relationships with study participants, as these organizations provide services to support these individuals.”

Phone calls were included to ensure participants that may not be as comfortable with the internet or texting would be able to hear about this opportunity. Advocacy organizations informed prospective participants about the focus group using any of the various approved communication means below (in-person, phone, email, text, and/or social media post), which has been added to the recruitment communication section of the supplemental materials. Phone calls utilized the same script as for in-person interactions:

Hello, thank you for your interest in participating in our study. My name is [Contact Person Name] and I am assisting a research study from Scripps Research Translational Institute to better understand any reasons why individuals that were formerly incarcerated would be willing and able to participate in research (ex. perspectives, time/availability, other activities or priorities). I would like to provide you with some information about the study and answer any questions you may have. Would you be interested in hearing more about participating in this study?

If no - Thank you for your time. Have a nice day/evening.

If yes - Great! Let me give you an overview of the study. If you are interested in participating, we will ask a few screening questions to check eligibility. If you are eligible and are selected, you may receive an invite to a focus group interview. This session will involve around 9 other people and will last about 75-120 minutes. In the focus group, we are interested in hearing your thoughts and experiences. We will ask you things like what you think research should do and what problems it should solve. The discussion might touch on your experiences before and after your time in incarceration and how that might influence your interest in research and science. The session will be audio-recorded on Zoom, but only for research purposes. We will keep all the information and recordings in a secure research database managed by trained staff. Plus, to protect your identity, you will get to pick pseudonyms (fake names). Your participation is completely voluntary and you can withdraw at any time without penalty. Based on the information provided, I’d like to confirm that you understand the details and agree to participate. Are you still interested in taking part?

If no - Thank you for your time. Have a great day/evening.

If yes - Great! Thank you for confirming your interest. We will proceed with next steps and provide you with further instructions.

Data analysis
It appears that a form of Thematic Analysis was used – but it’s not clear which model or approach? The description of the process seems a little confused. You state that ‘transcripts were reviewed to identify and label key themes, ideas, and patterns’ but surely you start with codes – which you seem to say in the latter sentence. You also state ‘themes, ideas, and patterns that emerge’ which is not in keeping with up to date thinking about Thematic Analysis. This section needs some reworking, and I’d suggest using citations and references to demonstrate the underpinnings of the approach
Author response: An initial review was conducted to identify themes, and then a second review was conducted to classify each component relative to themes. Braune & Clarke’s coding framework was utilized starting with familiarization. The data analysis section has been updated from page 8-10 to clarify the type of analysis framework utilized along with additional supporting references.

Cross-group synthesis
Again it is unclear where this approach to analysis has come from, citations and references to this method of analysis would be useful. Whilst I appreciate this is a synthesis the compare and contrast across groups sits less comfortably with qualitative work and philosophy – providing citations and references would help create a more robust explanation.

Author response: The data analysis section has been updated from page 8-10 to clarify the type of analysis framework utilized along with additional supporting references.
