# Supplementary material for: Re-imagining health research to include the voices of justice-impacted individuals
Source: PLOS Glob Public Health. 2026 Mar 3;6(3):e0006069. doi: 10.1371/journal.pgph.0006069 (PMC12956120; doi:10.1371/journal.pgph.0006069)
Supplement: Attachment — Submitted filename: review of Re-imagining health research.docx [file pgph.0006069.s008.docx]

## PLOS Global Public Health

## Re-imagining health research to include the voices of justice-impacted individuals

## Manuscript Number: PGPH-D-25-00414R2

Thank you for this important piece of research! It is clearly an important topic, and I appreciate your work. Because this is another round of review and the work is important, I have been quite comprehensive in my recommendations. I hope that they will be useful in getting this piece successfully published.

General

For future articles, please provide line numbers to aid with review.

Your methods section is lengthy, and your TA approach is not coherent – it seems you actually used a codebook type approach (framework analysis or template analysis), but you have cited Braun and Clarke’s reflexive TA. Needs revision.

Your results sections is a little sparse and not always well-supported by quotes (for example, the last paragraph). This section should be prioritised for depth and word count – it is the purpose of this article!

Your discussion section needs to better link your own findings to the existing literature and research, showing what you add and its importance/relevance.

Abstract

Paragraph 2 – change ‘the nation’ to ‘the United States’.

Introduction

First paragraph and throughout – make sure ‘US’ is used after acronym introduced. See also final paragraph introduction.

Second paragraph

- “With growing incarceration, there are also significant disparities.” – this sentence is not clear/complete and could be improved. Consider: “With increasing incarceration, there are also growing inequities.” Or “While incarceration has increased throughout the US, these increases are not equitably distributed.”
- “Black men and women are five times more likely to be incarcerated than White men, and Hispanic men are 1.3 times more likely (8,9).” – not a complete sentence. Hispanic men are 1.3 times more likely than what/who? Could rephrase: “Black men and women are five times more likely, and Hispanic men are 1.3 times more likely, to be incarcerated than White men.

Para 3

- “There are severe, immediate and long standing racial and socioeconomic disparities with individuals who are incarcerated that negatively affect mental and physical health and significantly impair successful reintegration.” - not clear. It is not necessarily the *disparity* that affects physical/mental health, so much as being incarcerated which creates the disparity… Consider rephrasing: “There are severe, immediate and long-term racial and socioeconomic disparities for justice-impacted individuals compared to others, including negative effects on their physical, mental, and social health which often significantly impairs their successful societal reintegration.”

Para 4

- “The effects extend beyond the individual to family members” – improve for clarity. Consider: “The negative effects of being justice-impacted also extends beyond the individuals to their family members”.
- “45% of people in the US have an immediate family member who is incarcerated” – don’t start the sentence with a number; write out as “Forty-five percent”

Final para

- “Individuals with previous US justice system involvement are one of the largest and most underserved populations in the world.” – In the world? Or in the US? If the world, provide reference, as this seems unlikely.

Methods

Recruitment

– must state (if true) that participation was voluntary

- Include the number of recruited participants

Study design

- You’ve mentioned that there were 5 advocacy groups, but how many focus groups were there in total? Did each advocacy group have one focus group? How many people per focus group?

Risks and Mitigation

Para 2

- What is “PII”?

Data analysis

- The transcription section should appear under the data collection / study design heading.
- You need to say who did what part of the analysis, not just “the research team”. Use acronyms for your names. Who coded the transcripts? If more than 1 person, how did you code and compare with multiple coders? Did all authors sit together to categorize codes into broader themes?
- It does not seem accurate to say your coding was purely inductive, as it seems you came with some pre-determined objectives about facilitators and barriers to research participation?
- If you did use Braun and Clarke’s TA (called reflexive TA) then you need to acknowledge your positionalities and their effects on the results.

Cross-Group synthesis

- You have now cited framework analysis - a distinct method of thematic analysis from Braun and Clarke’s – that is a type of codebook analysis. I suggest engaging with *Braun V, Clarke V. Toward good practice in thematic analysis: Avoiding common problems and be(com)ing a knowing researcher. International Journal of Transgender Health. 2023;24(1):1–6. DOI: 10.1080/26895269.2022.2129597*  and *Braun V, Clarke V. One size fits all? What counts as quality practice in (reflexive) thematic analysis? Qual Res Psychol. 2020;18(3):1–25. DOI: 10.1080/14780887.2020.1769238*. It seems that your overall TA method was more aligned to codebook analysis?

Results

Participants

- Consistency in capitalising race terms

Focus group results

- “Each synopsis statement below is followed by quotes from the participants on that topic, adapted to maintain the grammatical integrity and remove identifiable linguistic features such as regional or accented speech:” – this has already been mentioned. Remove.
- Need theme headings, with results grouped accordingly.
- Need identifier/pseudonym/focus group number after each quote
- “85% were interested in learning about their ancestry.” – not so much ‘learning about their ancestry’ (which could mean a host of things including social and cultural), as learning about health risks inherited from their ancestors?
- The last paragraph is not supported with quotes.
- In general, more needs to be made of this section.

Discussion

- I recommend starting your discussion with a very brief reminder of your research aim/s, and what you found.
- The first paragraph is largely a repetition of what is said in the introduction and is not directly relevant to your findings.
- Second paragraph is good as it compares your findings to other similar research. Discussion needs to be more in this vein, showing where your findings are novel or similar.
- As above, you need to more specifically discuss your own findings in relation to the literature. For example, where you say: “Collaboration with advocacy organizations that are supporting justice-impacted individuals can help studies ensure cultural competency and garner trust.” – while this may be true, you have not mentioned cultural competency or trust of advocacy groups in your results, and appears to come from nowhere.
- Final sentence: “We hope this shows it is feasible to include the participation of justice-impacted individuals in research studies.” – your study is not about feasibility and does not prove it is feasible to include these individuals. It *does* show that it is important, however, and you offer recommendations for improving participation. Revise final sentence to better reflect your study.

Thank you.
