# Supplementary material for: Re-imagining health research to include the voices of justice-impacted individuals
Source: PLOS Glob Public Health. 2026 Mar 3;6(3):e0006069. doi: 10.1371/journal.pgph.0006069 (PMC12956120; doi:10.1371/journal.pgph.0006069)
Supplement: Attachment — Submitted filename: RC response #4 to PLoS GPH.docx [file pgph.0006069.s009.docx]

Reviewer’s feedback on submission PGPH-D-25-00078, and author response.

Feedback Received on January 5, 2026

**Additional Editor Comments (if provided):**

Please restructure your results. Your quotes should not be presented as bullets, and they should be better integrated into the content of your results. I also see that a previous reviewer made this comment, so please ensure that you take this step in your next revision - thanks!
Author response: We have updated the format of the quotes and worked to better integrate them with the text.

**Reviewers' comments:**

Reviewer #3: I think that a plan on what the researchers are going to do after analyzing their findings in terms of policy changes, asking ethics bodies to make changes to make sure previously incarcerated people are not classified as vulnerable populations any more needs to be done for such a research to be of value to the community.

Author response: Thank you, we have added the following “We recommend that future work explores feasibility and implementation of meaningful inclusion of justice-impacted individuals.”

Reviewer #4: Thank you for this important piece of research! It is clearly an important topic, and I appreciate your work. Because this is another round of review and the work is important, I have been quite comprehensive in my recommendations. I hope that they will be useful in getting this piece successfully published.

Author response: Thank you for your thorough review of the manuscript. We respond in detail below.

*General*
For future articles, please provide line numbers to aid with review.

Author response: Our apologies for forgetting to add line numbers previously; we have added line numbers in this version.

Your methods section is lengthy, and your TA approach is not coherent – it seems you actually used a codebook type approach (framework analysis or template analysis), but you have cited Braun and Clarke’s reflexive TA. Needs revision.
Author response: The Data Analysis section has been revised on pages 8 and 9 to clarify that the study used an applied thematic analysis approach for multi-site health research. The language describing coding, consensus and theme development was updated to better reflect this framework.

Your results sections is a little sparse and not always well-supported by quotes (for example, the last paragraph). This section should be prioritised for depth and word count – it is the purpose of this article!

Author response: The results section has been revised to add quotes where needed and integrate the quotes with the other text in the section.

Your discussion section needs to better link your own findings to the existing literature and research, showing what you add and its importance/relevance.
Author response: We have added new content and references to the discussion along with removing duplicative content; more details are in the sections with specific suggestions below.

*Abstract*
Paragraph 2 – change ‘the nation’ to ‘the United States’.

Author response: This edit has been included.

Introduction
First paragraph and throughout – make sure ‘US’ is used after acronym introduced. See also final paragraph introduction.

Author response: This has been updated; note we left both instances of “United States” in the abstract since we did not think it was worth abbreviating for one additional use.

*Second paragraph*

- “With growing incarceration, there are also significant disparities.” – this sentence is not clear/complete and could be improved. Consider: “With increasing incarceration, there are also growing inequities.” Or “While incarceration has increased throughout the US, these increases are not equitably distributed.”

Author response: Your first suggestion was implemented.

- “Black men and women are five times more likely to be incarcerated than White men, and Hispanic men are 1.3 times more likely (8,9).” – not a complete sentence. Hispanic men are 1.3 times more likely than what/who? Could rephrase: “Black men and women are five times more likely, and Hispanic men are 1.3 times more likely, to be incarcerated than White men.
Author response: This suggestion has been implemented.

*Para 3*
- “There are severe, immediate and long standing racial and socioeconomic disparities with individuals who are incarcerated that negatively affect mental and physical health and significantly impair successful reintegration.” - not clear. It is not necessarily the disparity that affects physical/mental health, so much as being incarcerated which creates the disparity… Consider rephrasing: “There are severe, immediate and long-term racial and socioeconomic disparities for justice-impacted individuals compared to others, including negative effects on their physical, mental, and social health which often significantly impairs their successful societal reintegration.”
Author response: This suggestion has been implemented.

*Para 4*
- “The effects extend beyond the individual to family members” – improve for clarity. Consider: “The negative effects of being justice-impacted also extends beyond the individuals to their family members”.

Author response: This suggestion has been implemented.

- “45% of people in the US have an immediate family member who is incarcerated” – don’t start the sentence with a number; write out as “Forty-five percent”

Author response: We updated the punctuation before this to a semicolon so that starting with a number is acceptable.

*Final para*
- “Individuals with previous US justice system involvement are one of the largest and most underserved populations in the world.” – In the world? Or in the US? If the world, provide reference, as this seems unlikely.
Author response: Updated to “in the US.”

*Methods*
Recruitment
– must state (if true) that participation was voluntary

Author response: We added that participation was voluntary.

- Include the number of recruited participants
Author response: We added the number of participants (67) to the recruitment section.

*Study design*
- You’ve mentioned that there were 5 advocacy groups, but how many focus groups were there in total? Did each advocacy group have one focus group? How many people per focus group?
Author response: Yes, each advocacy group had its own focus group; this has been clarified.

*Risks and Mitigation*

*Para 2*
- What is “PII”?

Author response: This abbreviation is now spelled out: personally identifying information.

*Data analysis*
- The transcription section should appear under the data collection / study design heading.

Author response: This paragraph has been moved as suggested.

- You need to say who did what part of the analysis, not just “the research team”. Use acronyms for your names. Who coded the transcripts? If more than 1 person, how did you code and compare with multiple coders? Did all authors sit together to categorize codes into broader themes?

Author response: This has been updated to note that author RFB completed these tasks.

- It does not seem accurate to say your coding was purely inductive, as it seems you came with some pre-determined objectives about facilitators and barriers to research participation?

Author response: As noted above, the Data Analysis section has been revised on pages 8 and 9 to clarify that the study used an applied thematic analysis approach for multi-site health research. The language describing coding, consensus and theme development was updated to better reflect this framework.

- If you did use Braun and Clarke’s TA (called reflexive TA) then you need to acknowledge your positionalities and their effects on the results.

Author response: As noted above, the Data Analysis section has been revised on pages 8 and 9 to clarify that the study used an applied thematic analysis approach for multi-site health research. The language describing

*Cross-Group synthesis*
- You have now cited framework analysis - a distinct method of thematic analysis from Braun and Clarke’s – that is a type of codebook analysis. I suggest engaging with Braun V, Clarke V. Toward good practice in thematic analysis: Avoiding common problems and be(com)ing a knowing researcher. International Journal of Transgender Health. 2023;24(1):1–6. DOI: 10.1080/26895269.2022.2129597 and Braun V, Clarke V. One size fits all? What counts as quality practice in (reflexive) thematic analysis? Qual Res Psychol. 2020;18(3):1–25. DOI: 10.1080/14780887.2020.1769238. It seems that your overall TA method was more aligned to codebook analysis?

Author response: As noted above, the Data Analysis section has been revised on pages 8 and 9 to clarify that the study used an applied thematic analysis approach for multi-site health research. The language describing

*Results*
Participants
- Consistency in capitalising race terms
Author response: This has been reviewed.

*Focus group results*
- “Each synopsis statement below is followed by quotes from the participants on that topic, adapted to maintain the grammatical integrity and remove identifiable linguistic features such as regional or accented speech:” – this has already been mentioned. Remove.
Author response: This has been reviewed.

- Need theme headings, with results grouped accordingly.

Author response: The following headings were added: Perspectives on Research, The Importance of Health, Identifying and Removing Barriers to Research Participation

The Role of Community Support, and Making Time and Providing Compensation.

- Need identifier/pseudonym/focus group number after each quote

Author response: Participant quotes are attributed using anonymized codes indicating the partner organization, focus group, and participant number (e.g., P3-FG2-P5). Partner organizations were assigned numerical identifiers to protect confidentiality, and participant numbers reflect order of speaking rather than any personal characteristic.

- “85% were interested in learning about their ancestry.” – not so much ‘learning about their ancestry’ (which could mean a host of things including social and cultural), as learning about health risks inherited from their ancestors?
Author response: This sentence has been updated to “Also, 85% were interested in learning about their genetic ancestry and any impact this has on their health.”

- The last paragraph is not supported with quotes.

Author response: The following quotes were added to support this paragraph (along with reorganizing the paragraph as requested elsewhere):

"I've spent all my adulthood incarcerated. So I haven't been available."

"I've been approached to participate in research, it is so difficult for me because the [research appointments] are on days and times that I have to be at work."

- In general, more needs to be made of this section.
Author response: We have substantially revised the results section and appreciate this feedback.

*Discussion*
- I recommend starting your discussion with a very brief reminder of your research aim/s, and what you found.

Author response: This section now begins “We conducted focus groups with the goal of learning from justice-impacted individuals about their interest in participating in research and any barriers to doing so. Our focus groups also revealed a desire to participate in research, in particular with the potential to positively impact them and the broader justice-impacted community.”

- The first paragraph is largely a repetition of what is said in the introduction and is not directly relevant to your findings.

Author response: This content has been removed.

- Second paragraph is good as it compares your findings to other similar research. Discussion needs to be more in this vein, showing where your findings are novel or similar.
Author response: We added the following context regarding partnership with community organizations: “This is consistent with the broader literature on utilizing community-based approaches to strengthen research”.

- As above, you need to more specifically discuss your own findings in relation to the literature. For example, where you say: “Collaboration with advocacy organizations that are supporting justice-impacted individuals can help studies ensure cultural competency and garner trust.” – while this may be true, you have not mentioned cultural competency or trust of advocacy groups in your results, and appears to come from nowhere.

Author response: We added the following to introduce this paragraph: “Community-based participatory research has a long history of improving health research quality”.

- Final sentence: “We hope this shows it is feasible to include the participation of justice-impacted individuals in research studies.” – your study is not about feasibility and does not prove it is feasible to include these individuals. It does show that it is important, however, and you offer recommendations for improving participation. Revise final sentence to better reflect your study.

Author response: This sentence has been updated to read “This study demonstrates the importance of including the participation of justice-impacted individuals in research studies.” and is now followed by additional content based on a recommendation from another reviewer.
