# Supplementary material for: Re-imagining health research to include the voices of justice-impacted individuals
Source: PLOS Glob Public Health. 2026 Mar 3;6(3):e0006069. doi: 10.1371/journal.pgph.0006069 (PMC12956120; doi:10.1371/journal.pgph.0006069)
Supplement: Attachment — Submitted filename: response RC Feb 9 2026.docx [file pgph.0006069.s010.docx]

Reviewer’s feedback on submission PGPH-D-25-00078, and author response.

Feedback Received on February 8, 2026

Reviewer #4: Well done on producing this manuscript. Minor recommendations: Move our inclusion criteria from study design to recruitment, and consider a brief summarising sentence of your results before your discussion section.

Author response: Thank you for this feedback. Inclusion criteria was moved to the recruitment section. We added the following at the end of the results section “Taken together, participants relayed an interest in participating in research, often with the goal of helping others with experiences similar to theirs, and desire to overcome the logistical barriers that often prevent them from participating.”

Note: We uploaded our figure to the NAAS test website and received a notification that it passed.
